# Fresh fruit consumption and risk of incident albuminuria among rural Chinese adults: A village-based prospective cohort study

**DOI:** 10.1371/journal.pone.0197917

**Published:** 2018-05-24

**Authors:** Jiangping Wen, Jie Hao, Ye Zhang, Yuanbo Liang, Sizhen Li, Fenghua Wang, Xinrong Duan, Xiaohui Yang, Kai Cao, Bingsong Wang, Xinxin Lu, Ningli Wang

**Affiliations:** 1 Department of Laboratory Medicine, Beijing Tongren Hospital, Capital Medical University, Beijing, China; 2 Beijing Tongren Eye Center, Beijing Tongren Hospital, Capital Medical University, Beijing, China; 3 Beijing Eye Institute, Beijing, China; 4 Clinical and Epidemiological Research Center, the Affiliated Eye Hospital of Wenzhou Medical University, Wenzhou, China; 5 Nanjing Aier Eye Hospital, Nanjing, China; Shanghai Diabetes Institute, CHINA

## Abstract

**Background:**

Recent studies showed that increased fresh fruit consumption is significantly associated with lower risks of diabetes, hypertension, and cardiovascular disease (CVD); other studies suggested that albuminuria is associated with diabetes, hypertension, and CVD. Therefore, we hypothesized that lower fresh fruit consumption is associated with higher risk of incident albuminuria among Chinese adults in rural areas, where fresh fruit consumption level is very low and prevalence of albuminuria is high.

**Methods:**

We tested the hypothesis in a village-based cohort study of 3574 participants aged ≥ 30 years from the Handan Eye Study conducted from 2006 to 2013. Albuminuria was defined as urinary albumin-to-creatinine ratio ≥ 30 mg/g.

**Results:**

Overall, 35.4% of the participants never or rarely consumed fresh fruits, and 33.9, 21.6, and 9.1% consumed fresh fruits 1–3 times/month, 1–2 times/week, and ≥ 3 times/week, respectively. During a median follow-up period of 5.6 years, albuminuria developed in 17.6% (n = 629) of the participants. Compared with participants who consumed fresh fruits ≥ 3 times/week, the multivariable adjustment odds ratios (ORs) for incident albuminuria associated with fruit consumption 1–2 times/week, 1–3 times/month, and no or rare consumption were 1.58 (95% confidence intervals (CI), 1.05–2.40), 1.74 (95% CI, 1.17–2.58), and 1.78 (95% CI, 1.20–2.64), respectively. After excluding participants with diabetes, the association remained significant.

**Conclusions:**

Lower fresh fruit consumption was significantly associated with higher risk of incident albuminuria, and fresh fruit consumption frequency could be an essential intervention target to prevent albuminuria in rural China.

## Introduction

Fresh fruits are rich in fiber, antioxidants, vitamins, and phytochemicals that may have health benefits. Recent epidemiologic studies showed that increased fresh fruit consumption is significantly associated with lower risks of diabetes [1–2], hypertension [3], major coronary events [4], and stroke [4–5] in developed and developing countries. Albuminuria, which is generally defined as urinary albumin-to-creatinine ratio (UACR) ≥ 30 mg/g, could be due to renal microvascular injury and may reflect endothelial dysfunction [6–8]. Moreover, albuminuria is associated with risk of chronic kidney disease (CKD) progression, acute kidney injury, end-stage renal disease, and all-cause and cardiovascular-related mortality in the general population and in populations with increased cardiovascular disease (CVD) risk [8–12]. Currently, several studies in the USA and China found that diabetes, hypertension, and CVD are risk factors for albuminuria [13–18]. Therefore, fresh fruit consumption frequency may be associated with the risk of developing albuminuria in the general population.

In the USA, the Multi-Ethnic Study of Atherosclerosis (MESA) reported that a dietary pattern rich in whole grains and fruits is associated with lower UACR [19]; however, another study found that a prudent dietary pattern (higher intake of fruits, vegetables, legumes, fish, poultry, and whole grains) and the Dietary Approach to Stop Hypertension (DASH) style dietary pattern (greater intake of vegetables, fruits, and whole grains) are not associated with microalbuminuria [20]. Currently, relevant evidence from developing countries, such as China, where people have totally different eating habits (i.e., fresh fruits consumption frequency is extremely low, fresh fruits are usually consumed raw as snacks, and vegetables are usually fried or stewed as main dishes) [2,4,21] is limited. In China, a recent national survey showed that the prevalence of diabetes and hypertension is lower in rural than in urban areas, whereas the prevalence of albuminuria is higher in rural than in urban areas [14]. Furthermore, the China Kadoorie Biobank Study reported that fresh fruit consumption frequency is lower in rural than in urban areas [2,4]. Hence, we hypothesized that lower fresh fruit consumption is associated with higher risk of albuminuria among Chinese adults in rural areas. We prospectively investigated the associations between fresh fruit consumption frequency and risk of incident albuminuria in a village-based sample of rural Chinese adults in Yongnian County, Hebei Province.

## Materials and methods

### Study population

We analyzed the longitudinal data from the Handan Eye Study (HES), which is a village-based, prospective cohort study designed to survey eye diseases and other health-related problems in non-institutionalized, community-dwelling individuals aged ≥ 30 years in Yongnian (a rural county of Handan and located approximately 500 km south of Beijing). Detailed information about the methods and procedures pertaining to the survey was reported previously [22]. The population in Yongnian County was approximately 830,000 in 2000 (80% engages in farming and 98% are of Han ethnicity). Per capita net income of the rural households in this region is 3468 Yuan (approximately 468 USD), which is similar to the average income (3587 Yuan or 484 USD) of the rural citizens of the People’s Republic of China [23]. This study was approved by the Ethics Committee of Beijing Tongren Hospital (approval number: TREC2006-22), and all study procedures adhered to the recommendations of the Declaration of Helsinki. Written informed consent was obtained from all participants. A stamp of the right forefinger was accepted as an alternative to a signature for those who could not read or write. This approach was also approved by the Ethics Committee.

**Fig 1:**
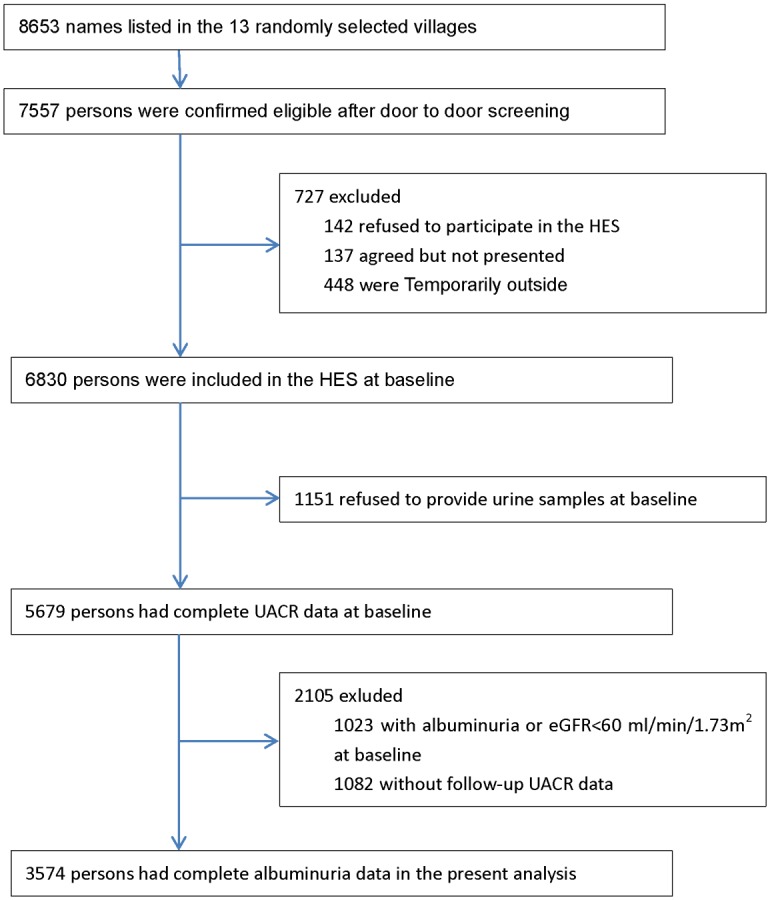
Flow diagram of recruitment of participants in the analysis.

Residents of Yongnian County, aged ≥ 30 years were randomly selected using a cluster sampling technique, with probabilities proportional to the size of the population in each cluster. Out of 453 villages, 13 villages in Yongnian County were selected to participate, wherein participants aged ≥ 50 years were selected; moreover, a random selection was also performed for those aged between 30 and 49 years in six of the 13 villages. As illustrated in Fig 1, of the 8653 individuals screened for HES, 7557 were eligible. A total of 6830 individuals participated in the baseline survey from October 2006 to October 2007, with a follow-up survey conducted between May 2012 and June 2013. At baseline, a total of 1151 individuals refused to provide urine samples and 1023 individuals with albuminuria (UACR ≥ 30 mg/g) or reduced renal function (estimated glomerular filtration rate (eGFR) < 60 mL/min/1.73 m^2^) were excluded. In the follow-up, 1082 individuals did not present with UACR data. Consequently, 3574 participants were included in the final analysis.

### Data collection

At the local study clinic, trained interviewers used questionnaires to obtain demographic information, including date of birth, sex, ethnicity, occupation, income, education, health status, health behavior (smoking, alcohol intake, and physical activity), diet, and medical history. Participant education was categorized into four groups according to the number of years of education (illiterate, 0 years; primary school, 1–6 years; middle school, 7–9 years; and high school or above, ≥ 10 years). Physical activity was classified as low (exercising rarely or never), moderate (walking or bicycling continuously for > 10 min, 1–4 times/week), and high (exercise resulting in rapid respiration for > 10 min, > 4 times/week). Dietary data included two major food groups: fresh fruits and fresh vegetables (S1 Diet-related questionnaires). Fresh fruit and vegetable consumption over the past year was classified into four frequency levels: ≥ 3 times/week, 1–2 times/week, 1–3 times/month, and never/rarely.

During the clinical examination, two blood pressure measurements were obtained with the participant in the seated position after 5 min of rest by using a non-invasive, automated Hem-907 blood pressure monitor (OMRON, Japan). Systolic blood pressure (SBP) and diastolic blood pressure (DBP) were calculated as the mean of two independent measurements. Body height and weight were measured with subjects not wearing shoes or outerwear. Height measurements were obtained using a wall-mounted measuring tape; weight measurements, using a bathroom scale (RGZ-120, China). Body mass index (BMI) was calculated as weight (kg) divided by height (m^2^). Waist circumference was measured at the mid-point between the last rib and the iliac crest in the mid-axillary line.

Urinary albumin and creatinine were tested from a fresh morning spot urine sample. Urinary albumin was measured by immunoturbidimetric methods (Diasys Diagnostic, Germany); urinary creatinine, by the enzymatic method (Shino-Test Corporation, Japan). Overnight fasting blood specimens were obtained for the measurement of creatinine, lipids, and glucose. Fasting plasma glucose (FPG) was measured by the hexokinase method and serum lipids by the enzymatic method (Shino-Test Corporation, Japan). Serum creatinine was measured using the same method as that used for urinary creatinine. eGFR was calculated with an equation developed by the adaptation of the Chronic Kidney Disease Epidemiology Collaboration (CKD-EPI) equation [24].

### Definition of albuminuria and reduced renal function

Albuminuria was defined as UACR ≥ 30 mg/g [8]. Reduced renal function was defined as an eGFR < 60 mL/min/1.73 m^2^, which is calculated as follows: eGFR = 141 × min(Scr/κ, 1)^α^ × max(Scr/κ, 1)^-1.209^ × 0.993^Age^ × 1.018 [if female], where Scr is serum creatinine concentration (in mg/dL) and age in years, κ is 0.7 for females and 0.9 for males, α is -0.329 for females and -0.411 for males, min is the minimum of Scr/κ or 1, and max is the maximum of Scr/κ or 1 [24].

### Definition of diabetes, hypertension, and CVD

Diabetes was defined as follows: FPG ≥ 7.0 mmol/L, self-reported diagnosis of diabetes, or use of antidiabetic medications [25]. Hypertension was positively identified if the individual had SBP ≥ 140 mmHg or DBP ≥ 90 mmHg or if antihypertensive medication was used [26]. CVD was defined by the presence of one or more of the following: self-reported diagnosis of coronary heart disease or stroke, or peripheral arterial disease, which was defined as an ankle-brachial index < 0.9 in either leg.

### Statistical analysis

This study included 3574 subjects who presented with complete albuminuria data (S1 Dataset). The baseline characteristics of the participants were described according to fruit consumption frequency, using means ± SD or median (interquartile range (IQR)) for continuous variables and counts and percentages for categorical variables. For comparisons of the mean or median values, analysis of variance or Kruskal-Wallis test was used. Categorical variables were analyzed using a χ^2^ test.

Non-conditional logistic regression was employed to estimate the odds ratios (ORs) and 95% confidence intervals (CI) of incident albuminuria by fresh fruit consumption frequency. We used two main multivariate models: model 1, adjusted for age and sex; model 2, included potential pathway intermediates (i.e., mediators on the causal pathway of the association between dietary intake and albuminuria), such as smoking, alcohol intake, regular consumption of fresh vegetables, BMI, waist circumference, physical activity, educational level, diabetes, hypertension, CVD, antihypertensive drug use, SBP, high-density lipoprotein-cholesterol (HDL-C), triglycerides, eGFR, and albumin-to-creatinine ratio.

In the sensitivity analysis, we repeated our primary analysis after excluding individuals with baseline diabetes. Furthermore, we repeated the analysis of the association between fresh fruit consumption and incident albuminuria using a sex-specific cutoff (i.e., UACR ≥ 17 mg/g for men and ≥ 25 mg/g for women) [27].

Statistical analysis was performed with SPSS (Statistical Package for the Social Sciences) v.18.0 software. A two-sided P value < 0.05 was considered statistically significant.

## Results

### Baseline characteristics

At baseline, the mean age was 50 years, 54.6% were women, 4.6% had diabetes, 44.8% had hypertension, and 7.7% had a history of CVD. Overall, 99.4% regularly consumed fresh vegetables, 35.4% never or rarely consumed fresh fruits, and 33.9, 21.6, and 9.1% consumed fresh fruits 1–3 times/month, 1–2 times/week, and ≥ 3 times/week, respectively (Table 1). A total of 629 individuals (17.6%) developed albuminuria and only 7 individuals developed reduced renal function (eGFR < 60 mL/min/1.73 m^2^) during the follow-up.

**Table 1:** Baseline characteristics of 3574 participants according to the frequency of fresh fruit consumption from the Handan Eye Study.

		Consumption of fresh fruit				
Characteristic	Overall (n = 3574)	≥3 times/week (n = 326)	1–2 times/week (n = 772)	1–3 times/month (n = 1210)	Never or rarely (n = 1266)	*P* value
Age, years	50 ± 11	47 ± 10	50 ± 10	50 ± 11	52 ± 10	<0.001
Female sex, n (%)	1951 (54.6)	189 (58.0)	441 (57.1)	632 (52.2)	689 (54.4)	0.100
BMI, kg/m^2^	24.6 ± 3.6	25.0 ± 3.3	24.9 ± 3.9	24.6 ± 3.7	24.4 ± 3.2	0.004
Waist circumference, cm	87.2 ± 9.3	86.0 ± 9.5	87.3 ± 9.4	87.1 ± 9.8	87.6 ± 8.8	0.060
SBP, mmHg	137.0 ± 20.9	134.3 ± 19.8	136.0 ± 19.5	136.4 ± 21.6	138.7 ± 21.1	0.001
DBP, mmHg	77.2 ± 11.7	77.0 ± 11.4	78.2 ± 11.7	76.9 ± 11.9	77 ± 11.7	0.090
Total cholesterol, mmol/L	4.57 ± 0.93	4.61 ± 0.99	4.55 ± 0.93	4.53 ± 0.91	4.60 ± 0.93	0.060
HDL-C, mmol/L	1.27 ± 0.28	1.27 ± 0.27	1.27 ± 0.27	1.26 ± 0.28	1.29 ± 0.29	0.060
LDL-C, mmol/L	2.68 ± 0.64	2.69 ± 0.65	2.67 ± 0.63	2.66 ± 0.64	2.70 ± 0.64	0.140
Triglycerides, mmol/L	1.25 (0.88–1.79)	1.29 (0.90–1.84)	1.28 (0.88–1.81)	1.26 (0.87–1.81)	1.20 (0.87–1.72)	0.230
FPG, mmol/L	5.49 (5.16–5.89)	5.50 (5.20–5.87)	5.45 (5.12–5.82)	5.50 (5.16–5.91)	5.51 (5.17–5.92)	0.110
Urea, mmol/L	4.78 ± 1.16	4.52 ± 1.09	4.78 ± 1.18	4.78 ± 1.16	4.84 ± 1.15	<0.001
Creatinine, μmol/L	70.8 ± 10.3	70.8 ± 10.9	70.4 ± 9.9	71.2 ± 10.6	70.7 ± 10.0	0.400
eGFR, ml/min/1.73 m^2^	96.3 ± 14.3	98.7 ± 13.6	96.8 ± 12.9	96.6 ± 14.9	95.2 ± 14.7	<0.001
Albumin-to-creatinine ratio, mg/g	6.89 (3.42–12.64)	7.12 (3.67–12.41)	6.88 (3.36–12.38)	6.69 (3.21–12.18)	7.20 (3.62–13.45)	0.180
Educational level, n (%)						
Illiterate	443 (12.4)	22 (6.7)	95 (12.3)	141 (11.7)	185 (14.6)	<0.001
Primary school	1849 (51.7)	138 (42.3)	392 (50.8)	607 (50.2)	712 (56.2)	
Middle school	1173 (32.8)	153 (46.9)	262 (33.9)	427 (35.3)	331 (26.1)	
High school or above	109 (3.0)	13 (4.0)	23 (3.0)	35 (2.9)	38 (3.0)	
Smoking, n (%)						
Never	2435 (68.1)	221 (67.8)	545 (70.6)	796 (65.8)	873 (69.0)	0.170
Previous	152 (4.3)	11 (3.4)	26 (3.4)	53 (4.4)	62 (4.9)	
Current	987 (27.6)	94 (28.8)	201 (26.0)	361 (29.8)	331 (26.1)	
Alcohol intake, n (%)						
Never	2785 (77.9)	233 (71.5)	613 (79.4)	917 (75.8)	1022 (80.7)	<0.001
Previous	106 (3.0)	4 (1.2)	20 (2.6)	39 (3.2)	43 (3.4)	
Current	683 (19.1)	89 (27.3)	139 (18.0)	254 (21.0)	201 (15.9)	
Physical activity, n (%)						
Low	644 (18.0)	56 (17.2)	77 (10.0)	241 (19.9)	270 (21.3)	<0.001
Moderate	299 (8.4)	59 (18.1)	24 (3.1)	64 (5.3)	152 (12.0)	
High	2631 (73.6)	211 (64.7)	671 (86.9)	905 (74.8)	844 (66.7)	
Diabetes, n (%)	165 (4.6)	10 (3.1)	26 (3.4)	59 (4.9)	70 (5.5)	0.070
Hypertension, n (%)	1602 (44.8)	115 (35.3)	352 (45.6)	527 (43.6)	608 (48.0)	<0.001
Cardiovascular disease, n (%)	276 (7.7)	23 (7.1)	58 (7.5)	89 (7.4)	106 (8.4)	0.750
Antihypertensive drugs use, n (%)	642 (18.0)	41 (12.6)	137 (17.7)	214 (17.7)	250 (19.7)	0.030
Regular consumption of fresh vegetables, n (%)	3551 (99.4)	321 (98.5)	771 (99.9)	1202 (99.3)	1257 (99.3)	0.060
Incident albuminuria, n (%)	629 (17.6)	36 (11.0)	132 (17.1)	219 (18.1)	242 (19.1)	0.007

Data are presented as mean±SD, median (interquartile range) or number (percent). *P* values are calculated using analysis of variance test or Kruskal-Wallis test for continuous variables and chi-square for categorical variables.

Participants who never or rarely consumed fresh fruits were older, more likely to be men, and had lower educational level at baseline compared with those who consumed fresh fruits ≥ 3 times/week. The proportion of participants with diabetes, hypertension, and abdominal obesity decreased as the frequency of fresh fruit consumption increased. The BMI and SBP of those who consumed fresh fruits ≥ 3 times/week were 0.6 points higher and 4.4 mm Hg lower, respectively, than those of individuals who never or rarely consumed fresh fruits. Baseline albumin-to-creatinine ratio, triglycerides, total cholesterol, HDL-C, and regular consumption of fresh vegetables were all similar across the frequencies of fresh fruit consumption.

### Association of baseline fresh fruit consumption with incident albuminuria

During a median follow-up period of 5.6 years, albuminuria developed in 17.6% (n = 629) of the 3574 participants. The risk of incident albuminuria was strongly and inversely associated with fruit consumption frequency (*P* < 0.001 for trend, for all comparisons) (Table 2).

**Table 2:** Odds ratios (95% confidence intervals) of incident albuminuria according to the frequency of fresh fruit consumption in 3574 participants.

Frequency of fresh fruit consumption	Number of individuals	Number of events	Odds ratio (95% CI)			
			Model 1	*P* value	Model 2	*P* value
≥3 times/week	326	36	1	Reference	1	Reference
1–2 times/week	772	132	1.58 (1.06–2.36)	0.024	1.58 (1.05–2.40)	0.030
1–3 times/month	1210	219	1.75 (1.20–2.56)	0.004	1.74 (1.17–2.58)	0.006
Never or rarely	1266	242	1.75 (1.19–2.56)	0.004	1.78 (1.20–2.64)	0.004
*P* for trend			0.02		0.01	

Model 1, adjusted for age and sex. Model 2, adjusted for age, sex, smoking, alcohol intake, regular consumption of fresh vegetables, BMI, waist circumference, physical activity, educational level, diabetes, hypertension, CVD, antihypertensive drugs use, SBP, total cholesterol, HDL-C, Triglycerides, eGFR, Albumin-to-creatinine ratio.

The age- and sex-adjusted OR for incident albuminuria was 1.58 (95% CI, 1.06–2.36) for those who consumed fresh fruits 1–2 times/week, 1.75 (95% CI, 1.20–2.56) for those who consumed fresh fruits 1–3 times/month, and 1.75 (95% CI, 1.19–2.56) for those who never or rarely consumed fresh fruits. After further adjustment for BMI, waist circumference, physical activity, educational level, diabetes, hypertension, CVD, antihypertensive drug use, SBP, eGFR, albumin-to-creatinine ratio, smoking, alcohol intake, regular consumption of fresh vegetables, triglycerides, total cholesterol, and HDL-C, the ORs for incident albuminuria were 1.58 (95% CI, 1.05–2.40) and 1.74 (95% CI, 1.17–2.58) for those who consumed fresh fruits 1–2 times/week and 1–3 times/month, respectively, and 1.78 (95% CI, 1.20–2.64) for those who never or rarely consumed fresh fruits. In our study, approximately 99.4% of participants reported daily consumption of fresh vegetables. Additional adjustment for consumption of fresh vegetables and other dietary variables did not alter the risk estimates for fresh fruit consumption.

### Sensitivity analyses

Initially, we repeated our primary analysis after participants with diabetes (n = 165) at baseline were excluded (Table 3). In the analysis, the association of fresh fruit consumption frequency with incident albuminuria remained significant (*P* value for trend < 0.05). In another sensitivity analysis, replacing the KDIGO (Kidney Disease: Improving Global Outcomes) definition of albuminuria with sex-specific definitions did not meaningfully change the association of fresh fruit consumption frequency with incident albuminuria (*P* value for trend = 0.001) (Table 4).

**Table 3:** Odds ratios (95% confidence intervals) of incident albuminuria according to the frequency of fresh fruit consumption in 3409 participants without diabetes.

Frequency of fresh fruit consumption	Number of individuals	Number of events	Odds ratio (95% CI)			
			Model 1	*P* values	Model 2	*P* values
≥3 times/week	316	35	1	Reference	1	Reference
1–2 times/week	746	118	1.46 (0.97–2.19)	0.069	1.43 (0.93–2.18)	0.101
1–3 times/month	1151	191	1.60 (1.09–2.36)	0.018	1.57 (1.05–2.35)	0.028
Never or rarely	1196	217	1.67 (1.13–2.46)	0.010	1.65 (1.10–2.46)	0.015
*P* for trend			0.02		0.02	

Model 1, adjusted for age and sex. Model 2, adjusted for age, sex, smoking, alcohol intake, regular consumption of fresh vegetables, BMI, waist circumference, physical activity, educational level, diabetes, hypertension, CVD, antihypertensive drugs use, SBP, HDL-C, Triglycerides, eGFR, Albumin-to-creatinine ratio.

**Table 4:** Odds ratios (95% confidence intervals) of incident albuminuria defined by a sex-specific cut-off according to the frequency of fresh fruit consumption in 3301 participants.

Frequency of fresh fruit consumption	Number of individuals	Number of events	Odds ratio (95% CI)			
			Model 1	*P* values	Model 2	*P* values
≥3 times/week	303	52	1	Reference	1	Reference
1–2 times/week	716	186	1.63 (1.16–2.30)	0.005	1.56 (1.09–2.24)	0.015
1–3 times/month	1119	302	1.72 (1.24–2.38)	0.001	1.64 (1.16–2.31)	0.005
Never or rarely	1163	345	1.89 (1.36–2.62)	<0.001	1.87 (1.33–2.63)	<0.001
*P* for trend			0.001		0.001	

Model 1, adjusted for age and sex. Model 2, adjusted for age, sex, smoking, alcohol intake, regular consumption of fresh vegetables, BMI, waist circumference, physical activity, educational level, diabetes, hypertension, CVD, antihypertensive drugs use, SBP, HDL-C, Triglycerides, eGFR, Albumin-to-creatinine ratio.

## Discussion

To our knowledge, this is the first study to investigate the relationship between fresh fruit consumption and incident albuminuria in the general Chinese population. In this village-based prospective cohort study with 5.6 years of follow-up, we found that lower fresh fruit consumption is significantly and independently associated with higher risk of incident albuminuria.

Several previous studies have investigated the associations of consumption of fruits and vegetables with risk of albuminuria and reported inconsistent findings in Western populations [19,20,28,29]. In a cross-sectional analysis of data from MESA including 5042 participants without clinical CVD and diabetes, Nettleton et al. [19] reported that a dietary pattern rich in whole fruits and vegetables is associated with lower UACR, and although not statistically significant, inverse associations between microalbuminuria and fruit consumption (OR, 0.94; 95% CI, 0.87–1.01) after adjustment for multiple demographic and lifestyle confounders were noted. Further, in an intervention study in the DASH Trial (greater intake of vegetables, fruits, and whole grains) involving 378 individuals without diabetes but with prehypertension or stage I hypertension, Jacobs Jr. et al. [28] confirmed that fruit/vegetable diet reduces urinary albumin excretion rate (AER) at 8 weeks in those with AER ≥ 7 mg/24 h at baseline. However, a subgroup analysis from the Nurses’ Health Study showed that the prudent and DASH style dietary patterns were not associated with microalbuminuria after multivariable adjustment [20]. These studies, which were conducted in Western populations, largely focused on dietary patterns and tended to combine fresh fruits, fresh vegetables, and other plant foods. The discrepancy among these studies could be attributed to the differences in the type of dietary pattern, sex, ethnicity, age, research design, presence or absence of diabetes, etc.

The eating habits of Chinese populations differ from those of Western populations. For example, in China, fresh fruit consumption frequency is lower. Fresh fruits are usually consumed raw as a snack, while fresh vegetables are usually fried or stewed as main dishes [2,4,21]. Moreover, to our knowledge, only the China Kadoorie Biobank Study reported associations between fresh fruit consumption frequency and microvascular complications in participants with diabetes [2]. In that study, Du et al. [2] found that those who consumed fresh fruits > 3 days/week has a significantly lower risk of developing diabetes-related microvascular complications (e.g., kidney diseases, eye diseases, and neuropathy) than those who consumed fresh fruits < 1 day/week; however, participants without diabetes were excluded and the definition of nephropathy was unclear. In our study, we focused on the relationship between fresh fruit consumption frequency and risk of incident albuminuria in the general Chinese population in rural areas. Consistent with our hypothesis, we found a robust association between fresh fruit consumption frequency and risk of incident albuminuria independent of several established chronic kidney disease risk factors, including diabetes, hypertension, and obesity.

Interestingly, similar to the results from the nationwide China Kadoorie Biobank Study [2], our findings revealed that the association between fresh fruit consumption frequency and risk of incident albuminuria is much stronger than that observed in Western populations [19,20,28]. Several possible reasons could explain this difference. In the rural Chinese population in our study, only 9.12% of the population consumed fresh fruits > 3 times/week, while 99.4% of the people consumed fresh vegetables daily. Therefore, the stronger association in our study could be attributed to the extremely low fruit consumption, which is supported by the non-linear dose-response association between fruit intake and disease risk in a previous study [30]. Moreover, the discrepancy between the studies could also be due to the differences in fruit types, frequency and type of animal food and dairy products, age, sex, ethnicity, research design, presence or absence of chronic diseases, such as diabetes and hypertension, etc. However, the exact mechanisms underlying the association of fresh fruit consumption with risk of incident albuminuria remain to be fully established. Nevertheless, fresh fruits are rich in fibers, antioxidants, vitamins, and phytochemicals, which in turn could provide several health benefits including antioxidative, anti-inflammatory, and antihypertensive effects and modulation of the composition and metabolic activity of gut microbiota, which could reduce the risk of vascular complications [2,31,32].

Our study has several limitations. First, a total of 6830 participants participated in the HES in 2006–2007, and the follow-up survey was conducted in 2012–2013. We excluded 3256 individuals for numerous reasons, thereby leaving 3574 participants for the final analysis. Thus, selection bias possibly exists. Second, we did not obtain information on the types of fruits consumed, which could vary greatly according to the season. Third, fruit consumption at baseline was assessed with a simple qualitative fruit-frequency questionnaire. Fourth, although 99.4% of our study population consumed fresh vegetables daily, we failed to properly examine the association of fresh vegetables with albuminuria risk. Finally, information on animal food (fat and meat), which has been suggested to be associated with the risk of hypertension, diabetes, and albuminuria, was not obtained. Although we have adjusted for total cholesterol, HDL-C, and triglycerides, residual confounding by animal food is still possible.

In conclusion, we found a robust association between fresh fruit consumption frequency and risk of incident albuminuria independent of several established risk factors for chronic kidney disease. Further studies including the types and quantities of fruits consumed among people living in rural and urban areas in China are required to confirm our findings and to elucidate the potential mechanisms underlying the association. Moreover, our findings suggested that particular aspects of diet, specifically fresh fruit consumption frequency, could be an essential intervention target to prevent albuminuria and subsequent kidney disease progression and CVD in rural China.

## Supporting information

S1 Diet-related questionnaires (DOCX)

S1 Dataset (SAV)
