# Supplementary material for: Fresh fruit consumption and risk of incident albuminuria among rural Chinese adults: A village-based prospective cohort study
Source: PLoS One. 2018 May 24;13(5):e0197917. doi: 10.1371/journal.pone.0197917 (PMC5967721; doi:10.1371/journal.pone.0197917)
Supplement: S1 Diet-related questionnaires — (DOCX) [file pone.0197917.s001.docx]

**Questionnaire 7**

**Participant I.D._______________ Gender：_————_ Age：_————————_ Date: _____/____/_____**

**Interviewer I.D._________**

| G13.  Fruit | How often do you eat fresh fruit?  1. daily  2. 3～6 times/week  3. 1～2 times/week  4. 1～3 times/month  5. <1 time/month | □ |
| --- | --- | --- |
| G14.  Vegetables | How often do you eat fresh vegetables?  1. daily  2. 3～6 times/week  3. 1～2 times/week  4. 1～3 times/month  5. <1 time/month | □ |
